# *N*-(3-Oxododecanoyl) Homoserine Lactone Is a Generalizable Plasma Membrane Lipid-Ordered Domain Modifier

**DOI:** 10.3389/fphys.2021.758458

**Published:** 2022-02-22

**Authors:** Hefei Ruan, Chunlin Zou, Yanni Xu, Xiaohong Fang, Tie Xia, Yan Shi

**Affiliations:** ^1^Beijing Key Lab for Immunological Research on Chronic Diseases, Department of Basic Medical Sciences, Tsinghua-Peking University Joint Center for Life Sciences, School of Medicine, Institute for Immunology, Tsinghua University, Beijing, China; ^2^Key Laboratory of Molecular Nanostructure and Nanotechnology, CAS Research/Education Center for Excellence in Molecular Sciences, Institute of Chemistry, Chinese Academy of Sciences, Beijing, China; ^3^University of Chinese Academy of Sciences, Beijing, China; ^4^Department of Microbiology, Immunology and Infectious Diseases, Snyder Institute, University of Calgary, Calgary, AB, Canada

**Keywords:** lipid raft, 3oc, MβCD, plasma membrane, cholesterol, lipid-ordered domain

## Abstract

A mammalian plasma membrane is a structure on which several layers of complexity are built. The first order of complexity comes from the heterogeneity of lipid-ordered domains. Gangliosides in concert with cholesterol are preferentially packed on the outer leaflet and form lipid-ordered domains, commonly known as lipid rafts. The formation and dynamics of these domains impact nearly all membrane protein functions and are an intensely studied topic. However, tools suited for lipid domain alteration are extremely limited. Currently, methyl-β-cyclodextrin (MβCD) appears to be the most common way to disrupt lipid domains, which is believed to operate *via* cholesterol extraction. This significantly limits our ability in membrane biophysics research. Previously, we found that *N*-(3-oxo-dodecanoyl) homoserine lactone (3oc), a small signaling chemical produced by *Pseudomonas aeruginosa*, is highly efficient in altering lipid-ordered domains. In this study, 3oc was compared with MβCD in a series of biochemical, biophysical, and cell biological analyses. Per molarity, 3oc is more efficient than MβCD in domain alteration and appears to better retain membrane lipids after treatment. This finding will provide an essential reagent in membrane biophysics research.

## Introduction

The eukaryotic plasma membrane is heterogeneous in lipid composition as well as in protein contents. It is recognized that this heterogeneity is critical for transmembrane receptor signaling (Simons and Toomre, 2000; Shi and Ruan, 2020). While the complexity is multidimensional, lipid-ordered domains are considered to be at the root whereby other heterogeneities are further added. It is generally accepted that glycosylated gangliosides, particularly sphingomyelin, are preferentially packed on the outer leaflet with positive curvature. Coupled to the repulsion of polyunsaturated phospholipids, the cone-shaped gangliosides are stabilized by small cholesterol to form a vertically slightly elevated area known as lipid-ordered domain (*Lo*, also known as lipid rafts) in reference with the rest of the membrane (*Ld* or lipid disordered domains) (García-Arribas et al., 2016; Wang et al., 2017). These domains are highly dynamic and are in constant transition of formation (demixing) and dissolution (mixing) (Kusumi et al., 2005). As they are under optical diffraction, indirect measurements have different estimations of their sizes, ranging from 20 to 200 nm (Pralle et al., 2000; Pike, 2006).

While the biochemical and biophysical characterization is ongoing, as proposed by Kai Simons, the role of lipid rafts in receptor signaling regulation has been confirmed in essentially all aspects of mammalian biology, particularly immunology, development, cancer, and neurology (Lee et al., 2010; Asakura et al., 2015; Nakayama et al., 2018; Vona et al., 2021). For instance, the components of the T cell receptor (TCR) complex are distributed either in or out of *Lo* at the resting state. TCR signaling triggers a reorganization of those components to form a large disk-like structure driven by lipid dynamics (Orbach and Su, 2020; Zhang et al., 2021). *Lo* domains also sort the lipid species on the inner leaflet which leads to the regulated exposure of secondary messengers and stabilizes the plasma membrane onto the cortical cytoskeleton (Mu et al., 2018).

One of the commonly used approaches in earlier years was to extract lipid rafts with cold non-ionic detergents (i.e., Triton X-100), which resulted in detergent-resistant membrane domains (DRMs). However, DRMs are increasingly regarded as imprecise representations of real-life lipid domains (Levental et al., 2020). Another approach is to extract cholesterol from the plasma membrane. Cyclodextrins are cyclic oligosaccharides that contain a hydrophobic core, with a hydrophilic outer surface. Their water solubility and affinity to membrane permit their use as a tool to extract cholesterol from the plasma membrane (Ohvo and Slotte, 1996; López et al., 2011). Cholesterol-saturated cyclodextrins can also be used as a shuttle to increase the cholesterol content in live-cell membranes. This technique has been the main staple in lipid domain research and is the technical basis for a lot of literature on lipid rafts. However, the technique is inefficient and time-consuming, requiring often more than one round of depletion. In our previous study, we found that the LasI-LasR circuit of quorum sensing in *Pseudomonas aeruginosa* relies on the production of *N*-(3-oxo-dodecanoyl) homoserine lactone (3oc) (Song et al., 2019). 3oc is essential for *P. aeruginosa* to inhibit host immune attack. Mechanistically, 3oc inserts itself into the lipid-ordered domains of host immune cells and causes their dissolution. This leads to the spontaneous trimerization of tumor necrosis factor receptor 1 (TNFR1), resulting in apoptotic cell death in host immune cells. 3oc is highly efficient, with a working concentration of low μM. In this report, we systematically compared 3oc to methyl-β-cyclodextrin (MβCD) as a generalizable *Lo* modifier. Results indicate that the 3oc is comparable to MβCD by several key parameters and with some beneficial characters and suits the needs for lipid domain research.

## Materials and Methods

### Cell Culture

Hela and COS-1 cells were cultured in Dulbecco’s Modified Eagle Medium (DMEM, Gibco), supplemented with 10% fetal bovine serum (FBS, HyClone), 100 mg ml^–1^ streptomycin, and 100 U ml^–1^ penicillin at 37^°^C in a 5% CO_2_ atmosphere. THP-1 cells were cultured in RPMI-1640 medium (HyClone), supplemented with 10% FBS, 100 U ml^–1^ penicillin, 100 mg ml^–1^ streptomycin, 10 mM HEPES, and 50 μM β-mercaptoethanol at 37^°^C in a 5% CO_2_ atmosphere.

### Giant Plasma Membrane Vesicle Isolation and Staining

Before giant plasma membrane vesicle (GPMV) isolation, live cells were incubated with DiD (1:1,000, Invitrogen, V22887), which is a lipophilic fluorescent dye located in the disordered domain, for 10 min at 37^°^C, then washed three times with “GPMV buffer” (10 mM HEPES, 150 mM NaCl, 2 mM CaCl_2_, pH 7.4), and then incubated at 37°C for about 60 min in GPMV buffer containing 25 mM PFA and 2 mM DTT to induce vesiculation (Levental et al., 2010). The supernatant was collected and incubated with 0, 1, 10, 30, 100, 200, 500 μM, and 1, 5, 10, 30 mM MβCD or 0, 1, 5, 10, 30 μM 3oc, and then used for three-dimensional (3D) Confocal imaging to analyze the distribution of ordered and disordered domains.

### Membrane Components Extraction

Hela cells were used to analyze the effect of MβCD (Sigma–Aldrich, C4555) and 3oc (Cayman, 10007895) on cell membrane components. For cholesterol extraction experiment, because the efficiency of inserting cholesterol into the cell membrane by itself is very low, in this study, we first formed a saturated solution of BODIPY-cholesterol (10 μg/ml, Avanti Polar Lipids, 810255P) and MβCD in DMEM and then used the MβCD to load cholesterol onto the cell membrane for 30 min. After the cells were washed two times with DMEM, they were treated two times with 10 mM MβCD or 10 μM 3oc for 30 min. For sphingomyelin (SM), phosphatidylcholine (PC), and phosphatidylethanolamine (PE) extraction experiment, fluorescent-labeled lipids NBD-SM (10 μg/ml, Avanti Polar Lipids, 810218P), TMR-PC (10 μg/ml, Avanti Polar Lipids, 810180P), and TMR-PE (10 μg/ml, Avanti Polar Lipids, 810241P) were used to label the cell membrane for 30 min, after the cells were washed two times with DMEM and then treated with 10 mM MβCD or 10 μM 3oc for 30 min. Finally, these samples were fixed and then used for total internal reflection fluorescence microscope (TIRFM) imaging to analyze the corresponding lipid composition changes on the cell membrane.

### Analysis of Endogenous Lipid Changes by Liquid Chromatography-Mass Spectrometry

Liquid chromatography-mass spectrometry (LC-MS) was used to measure changes in endogenous lipid composition. Hela cells were seeded in a 10-cm cell culture dish for 24 h; before treatments, the cells were washed two times with phosphate-buffered saline (PBS) to remove cell debris and dead cells and then treated with 10 mM or 10 μM 3oc (both MβCD and 3oc were diluted with PBS) for 30 min. Cell supernatants were extracted two times with a mixture of dichloromethane and methanol (volume ratio 2:1). After drying with nitrogen, they were reconstituted with a mixture of dichloromethane and isopropanol (volume ratio 1:4), and then corresponding internal standards were added for LC-MS analysis. Among them, the concentration of cholesterol is calculated using cholesterol standards, and other lipids are calculated using isotope-labeled internal standards 18:1 (d9) SM, 15:0–18:1 (d7) PC, and 15:0–18:1 (d7) PE (Avanti Polar Lipids, 791649, 791637, and 791638, respectively). For cholesterol, LC-MS was performed on the AB SCIEX QTRAP 4500 (United States) triple quadrupole mass spectrometer in SRM and positive ionization mode. The LC separation was run on an XBridge BEH C18 column. Methanol and isopropanol (8:2) containing 0.5 mM amine acetate were as solvent A, and water containing 0.5 mM ammonium acetate as solvent B. The MS parameters are as follows: APCI ion source temperature 400°C; air curtain: 30 psi; collision activated dissociation (CAD) gas settings: medium, ion spray voltage: 5,500 V; and ion gas 1: 40 psi; all data use Analyst 1.6.3 Software analysis, and the optimized cholesterol MRM acquisition mode is the dehydration peak (M/Z 369.4 > 161.2; 369.4 > 147.3; CE = 30 V) to draw a standard curve of cholesterol to calculate the content of the sample. For other lipids, LC-MS was performed on the AB SCIEX QTRAP 5500 (United States) triple quadrupole mass spectrometer, in SRM and positive and negative ionization modes. The LC separation was performed on an XBridge BEH C8 column using 70% acetonitrile aqueous solution containing 5 mM ammonium acetate as solvent A and 5 mM isopropanol as solvent B. The MS parameters are as follows: ESI ion source temperature 500°C; air curtain: 30 psi; CAD gas settings: medium; ion spray voltage: 5,500 V/-4,500 V; ion gases 1 and 2: 50 psi. The data acquisition software is AB SCIEX Analyst 1.7.1, and the data analysis software is SCIEX OS-MQ 1.6.1.

### Atomic Force Spectroscopy of Cell Membranes

Force measurements were performed by using a commercial atomic force microscope (Cellhesion200 from JPK Instrument, Berlin), which was mounted on top of the inverted optical microscope (Zeiss Axio Observer D1). A soft cantilever (nominal spring constant: 0.011 N/m, HYDRA2R-100NG, ApppNano, United States) with no tip modification was used to touch and pull the cell membrane tethers. The applied force was 0.5 nN, and the contact time was 5 s in constant height mode. The pulling rate is 10 μm/s. Cells were seeded on the glass substrate in the culture medium 1 day before the force measurements. Typically about 50 cells were probed in each condition, and each cell was probed 3 times. The force curves were analyzed with JPK data processing software, and the last tether breaking force (Ft) was used to compute the apparent membrane tension (T) based on the following formula: $T=\frac{F_{t}^{2}}{8\,\pi^{2}\,K}$, where *K* is membrane bending stiffness and is about 3 × 10^–19^ N/m on average (Sheetz, 2001).

Membrane tension of Hela cells in resting state and on treatment of 3oc and MβCD, respectively, was computated from the tether force measurements according to the abovementioned formula. For 3oc and MβCD experiments, force probing was started after the 10-min incubation. Each data point was from one force measurement, and for each cell, 3 measurements were performed, and about 50 cells were probed for each experimental condition.

### Endocytosis of TLR4

The THP-1 cells were used to study the effect of MβCD and 3oc on lipid raft-dependent endocytosis of TLR4. THP-1 cells were first treated with 10 μM Chlorpromazine Hydrochloride (CPZ, Sigma-Aldrich, C0982) for 24 h and then treated with 10 mM MβCD for 30 min or 10 μM 3oc for 10 min, and then 10 μg/ml LPS was used to induce the endocytosis of TLR4 for 30 min. Finally, the cells were fixed and then stained with primary antibody of TLR4 (Abcam, ab22048) and secondary antibody of anti-mouse Alexa 488 (Invitrogen, A-11001) for flow cytometry analysis.

### Confocal Imaging

The cortical cytoskeleton imaging experiments were performed on Hela cells. Hela cells were seeded in a 35-mm confocal dish 24 h before experiments, and then the cells were treated with 10 mM MβCD or 10 μM 3oc for 0, 10, 30, 60, 120, and 240 min. After the cells were fixed, Phalloidin-Alexa Fluor 568 (1:200, Invitrogen, A12380) and DAPI (1:1,000) were used to stain the cytoskeleton and nucleus separately. Finally, the confocal imaging was performed, and ImageJ was used for the statistical analysis of average fluorescence intensity.

The 3D imaging of GPMVs was performed at room temperature by a commercial Spinning Disk Confocal Microscope (Andor Dragonfly, Oxford Instruments) equipped with a 100 × 1.45 NA objective. During imaging, DiD was excited by a 647-nm laser, and the emission was collected by using a 660–700 nm band-pass filter. The 3D imaging was recorded with 2,048 × 2,048 pixels and with a step size of 200 nm. The two-dimensional (2D) and Z-stack images were processed by ImageJ “Z project” plugin.

### Total Internal Reflection Fluorescence Microscope Imaging

The abundance of cholesterol (Chol), SM, PC, and PE on the cell membrane was analyzed by a commercial TIRFM (Nikon TiE Inverted Microscope) equipped with a 100 × 1.49 NA objective and an Andor iXon DU897 EMCCD. During imaging, BODIPY-cholesterol and NBD-SM were excited by a 488-nm laser, and the emission was collected by using a 500–550 nm band-pass filter. TMR-PC and TMR-PE were excited by a 561-nm laser, and the emission was collected by using a 580–620 nm band-pass filter. After the respective process, we imaged the fluorescence intensity of the bottom surface of each group of cells and then used the ImageJ program to perform statistical analysis (about 30 cells per group).

### Epidermal Growth Factor Receptor and Extracellular Signal-Regulated Kinase Activation

The COS-1 cells were seeded in 6-well plates before treatment to 70% confluency, and the media were switched to serum-free DMEM. Then, COS-1 cells were incubated with MβCD or 3oc that dissolved in serum-free DMEM for the indicated times at 37°C. After treatment, cells were washed with cold PBS and then lysed with RIPA buffer (Beyotime; P0013B). Cell lysates were centrifuged at 12,000 rpm, 4°C for 10 min in an Eppendorf centrifuge, and the supernatants were collected. After boiling in 5 × SDS sample buffer for 10 min, the supernatants were run on 10% acrylamide gel for 90 min at 90 V and then transferred to nitrocellulose filter membrane (PALL; 66485) at 260 mA for 90 min. For immunoblotting, the nitrocellulose filter membrane was blocked with 5% milk in Tris-buffered saline Tween-20 (TBS-T). After incubation with a specific primary antibody in primary antibody dilution buffer (Beyotime; P0256) overnight at 4°C, the membrane was washed in TBS-T for 4 times and each time for 5 min. Horseradish peroxidase (HRP)-conjugated secondary antibodies were diluted in TBS-T and incubated for 1 h at room temperature. Then, positive immune reactive signals were detected by Amersham Imager 600 in the detection reagent (Yeasen; 36208ES76). The antibodies against epidermal growth factor receptor (EGFR, 4267), phosphorylated EGFR (pEGFR, 4407), extracellular signal-regulated kinase (ERK, 9102), phosphorylated ERK (pERK, 9101), and HRP-conjugated anti-mouse (7076) or rabbit (7074) secondary antibody were from CST.

### Caspase Activation

The THP-1 cells were diluted to 0.75 × 10^6^ cells/ml and seeded in 6-well plates. Then, the cells were treated with 3oc for the indicated times at 37°C. After treatment, cells were collected and centrifuged at 3,000 rpm for 3 min at 4°C, and then cell pellets were lysed. Cell lysates were applied to the Western blots as mentioned earlier, but samples were run on 12.5% acrylamide gel. Antibodies against Caspase 3 (9665), cleaved Caspase 3 (9664), and GAPDH (51332) were from CST.

### Apoptosis

The THP-1 cell apoptosis was detected by the Annexin V/PI Apoptosis Detection Kit (4A Biotech; FXP023-100), following the user guide. In brief, cells were diluted to 0.75 × 10^6^ cells/ml and seeded in a 12-well plate and then stimulated with 3oc for the indicated concentrations. Notably, 5 h later, cells were collected and stained with Annexin V-FITC and PI, and then the apoptotic effect was measured by flow cytometry. To normalize for spontaneous cell death, the percentage of live cells was calculated as follows: Live cell% = (Annexin V negative cells in MβCD or 3oc treatment/total cell)/(Annexin V negative cells in DMSO treatment/total cells) × 100%.

## Results

### 3-Oxododecanoyl and MβCD Treatments Induce Different Lipid-Ordered Domain Alterations

**FIGURE 1:**
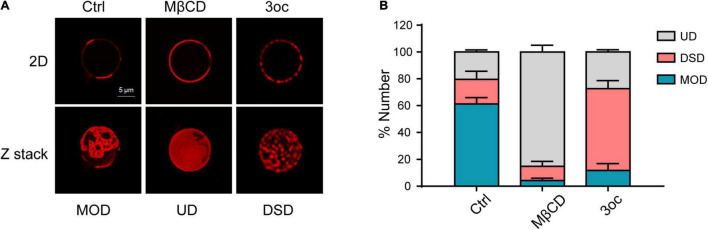
Characterizing the effects of methyl-β-cyclodextrin (MβCD) and *N*-(3-oxo-dodecanoyl) homoserine lactone (3oc) treatments on lipid-ordered domain on giant plasma membrane vesicles (GPMVs). **(A)** The upper panels show the distribution of ordered domains at the maximum diameter of GPMVs. Images from left to right are control, MβCD (10 mM), and 3oc (10 μM) treatments. The lower panels are the corresponding Z-stack images. **(B)** Statistical results show that mixed ordered and disordered (MOD) (59%) dominates the untreated GPMVs, MβCD, and 3oc treatment preferentially resulted in uniform disordered (UD) (86.6%) and disintegrated small domain (DSD) (63.05%), respectively. This experiment was repeated three times, each with 40–50 vesicles per condition. Scale bar = 5 μm.

As live-cell lipid-ordered domains are below optical diffraction, and their disruption causes a significant change in morphology and signaling transduction, we decided to use GPMVs to characterize the effects of MβCD and 3oc treatments, as they contain lipid domains observable under the conventional microscope in the absence of cellular activities (Baumgart et al., 2007). Using DiD to reveal disordered domains, Figure 1 shows GPMV from Hela cells displayed patch-like lipid-ordered domains without treatment, which are larger than those forming on the live-cell membrane (Pralle et al., 2000; Pike, 2006). From the Z-stack, the DiD label-free areas were round, likely representing the gel phase in the presence of SM and cholesterol. MβCD and 3oc treatments both removed those structures, in 2D view or in Z-stack. Interestingly, MβCD and 3oc treatments resulted in very distinct domain alterations. MβCD produced a more homogeneous surface, likely due to the extraction of cholesterol leading to mixing (diffusion) of the lipophilic fluorescent dye DiD. Some “gel” phases with irregular surrounds remained, which might be a different type of domain occurring in the absence of cholesterol (Bagatolli, 2006). In contrast, 3oc produced a more limited disintegration with small DiD islands remaining (Figure 1A and Supplementary Movies 1–3). We categorized GPMV into three types: One, represented by the control treatment with 2–5 continuous DiD phases per vesicle in a 2D image, was termed mixed ordered and disordered (MOD). The second, represented by MβCD treatment, with total DiD labeling of the entire circumference, was termed uniform disordered (UD), and the third, represented by 3oc treatment, with more than 5 distinct DiD label areas, was termed disintegrated small domains (DSDs). Figure 1B shows that in untreated GPMV, MOD dominated as expected. However, MβCD and 3oc preferentially resulted in UD and DSD, respectively. We also compared the concentrations of MβCD and 3oc required to achieve membrane alteration. 3oc showed an initial effective concentration around 5 μM with DSD features more apparent in 10 and 30 μM. In contrast, MβCD led to a UD-dominated feature around 100 μM. In no circumstance, were DSD and UD dominant with MβCD and 3oc treatments, respectively (Supplementary Figure 1). From these results, it appeared that both 3oc and MβCD alter lipid-ordered domains, but their mechanisms are different.

### 3-Oxododecanoyl Disrupts Lipid-Ordered Domains Without Changing Plasma Membrane Components

**FIGURE 2:**
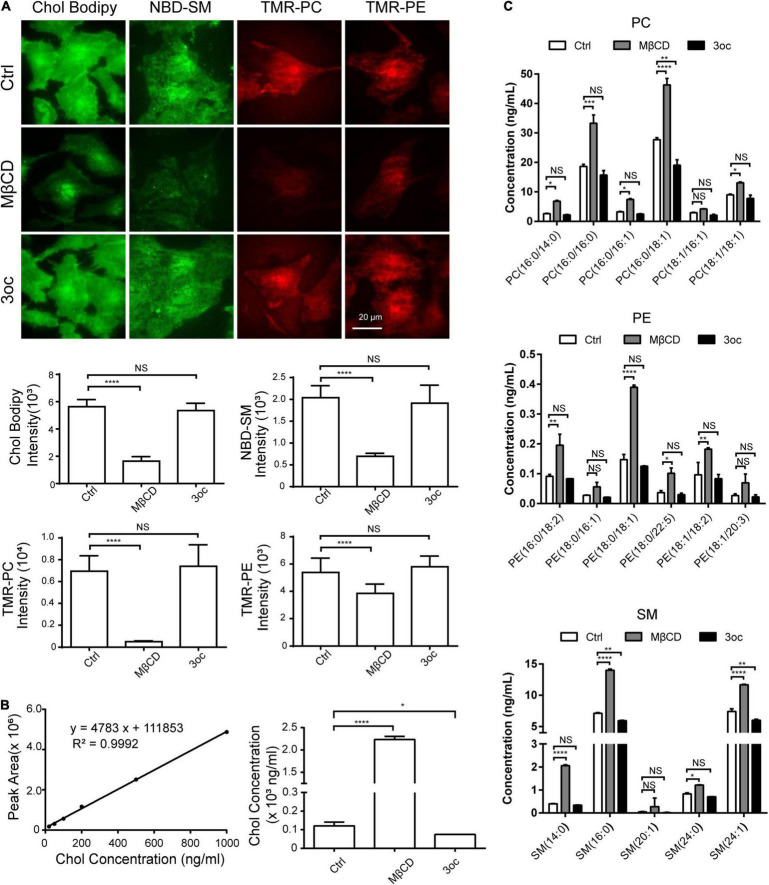
Effects of MβCD and 3oc treatments on cell membrane components. **(A)** Fluorescently labeled cholesterol (Chol), sphingomyelin (SM), phosphatidylcholine (PC), and phosphatidylethanolamine (PE) are used to analyze the effects of total internal reflection fluorescence microscope (TIRFM) imaging. From left to right, BODIPY-Chol, NBD-SM, TMR-PC, and TMR-PE are used to label the Hela cell membrane. The histograms below are the average fluorescence intensity of about 30 cells in each group. Significant differences were analyzed by an unpaired *t*-test. **(B)** The concentrations of endogenous cholesterol in cell supernatant after MβCD and 3oc treatments were analyzed by liquid chromatography-mass spectrometry (LC-MS), the left panel is the cholesterol standard curve, and the right panel is the calculated results. **(C)** The concentrations of endogenous PC, PE, and SM in cell supernatant after MβCD and 3oc treatments were analyzed by LC-MS, from top to bottom are the concentration changes of PC, PE, and SM. Significant differences were analyzed by a two-way ANOVA test. Scale bar = 20 μm. Henceforth, *, **, ***, and ****, *P* < 0.05, 0.01, 0.001, and 0.0001, respectively.

As MβCD is known to extract cholesterol from the bilayer membrane, we labeled cells with fluorescent Chol, SM, PC, and PE and measured their abundance following the two different treatments with total internal reflection fluorescence (TIRF) imaging. MβCD reduced cholesterol label, while 3oc did not. Remarkably, despite the proposed cholesterol specificity of MβCD, its treatment removed all four lipid species with nearly identical efficacy, while 3oc showed no such depletion (Figure 2A). Although the addition of labeled lipids into the membrane did not cause an observable increase in cell death (Supplementary Figure 2), this experiment was unlikely to completely mimic the real lipid dynamics, as all three labels, namely, BODIPY, NBD, and TMR, are of considerable molecular weights and are attached to targeted species at different positions. To avoid artifacts related to the labeling, we decided to use internally controlled LC-MS to measure all the relevant lipids. Cholesterol LC absorption scan peak area was almost linear to sample amounts loaded, thus allowing the production of a standard curve. For treated samples, the amount of cholesterol present in the salutation phase was determined *via* curve fitting and it was found to be about 20-fold higher than the control while 3oc did not show any increase (Figure 2B). Using isotope-labeled 18:1 (d9) SM, 15:0–18:1 (d7) PC, and 15:0–18:1 (d7) PE as internal ionization references, we performed LC-MS to measure the amounts of SM, PC, and PE of common acyl chain lengths (∼14–24) and saturation variants (Figure 2C). While these variants did not completely cover all possible acyl chain lengths of plasma membrane lipids, the general trend was clear that compared with 3oc, MβCD was uniquely capable of extracting all lipid species tested. This analysis of native lipid species gave a conclusion similar to Figure 2A. Regarding its cell biological impact, as plasma membrane lipid abundance is quantitatively maintained, MβCD is expected to cause significant replenishment of membrane lipids, an effect spared by 3oc. As accelerated plasma membrane lipid turnover alters the cell biological properties from several angles, particularly the lipid domain formation and signaling half-lives of surface receptors (Casares et al., 2019), we suggest additional caution in interpreting the cell signal change as MβCD treatment results in more membrane behavior changes than what is associated with cholesterol extraction alone.

### 3-Oxododecanoyl and MβCD Treatments Lead to a Similar Reduction in Membrane Tension

**FIGURE 3:**
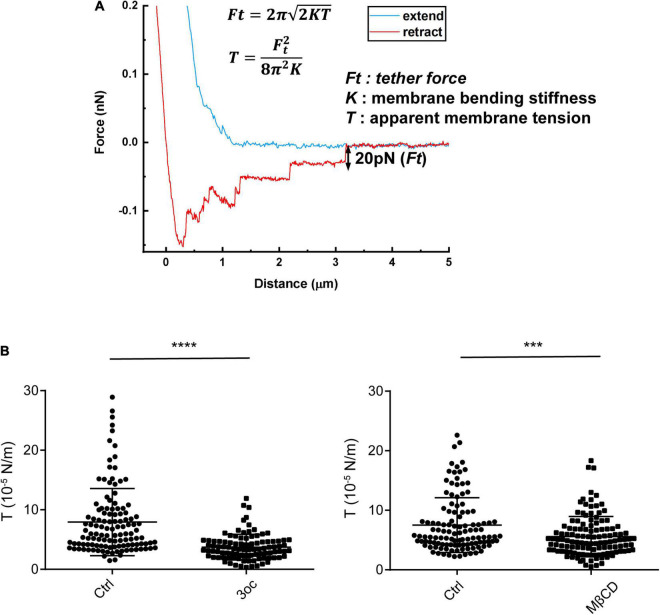
Membrane tension probed by atomic force microscopy (AFM)-based tether force measurements. **(A)** A typical force curve showing membrane tethers was pulled out from the cell membrane and broken in a stepwise manner. The last step size (i.e., tether force, Ft) was used to compute apparent membrane tension (T), based on the formula (inset), where *K* is membrane bending stiffness and is about 3 × 10^– 19^ N/m on average. **(B)** Membrane tension of Hela cells in resting state and after the treatment of 3oc and MβCD, respectively. For 3oc and MβCD experiments, force probing was started after the 15-min incubation. Each data point was from one force measurement, and for each cell, 3 measurements were performed and about 50 cells were probed for each experimental condition. ****P < 0.0001 and ***P < 0.0003 as measured by an unpaired *t*-test.

In our previous study, it was found that 3oc causes a significant reduction in membrane packing (Song et al., 2019). This is expected to cause a reduction in tether force, an important property in cell or cell surface contact (Pontes et al., 2017). To quantify this alteration and compare the effects of the two treatments, we used atomic force microscopy (AFM) to analyze the force required to complete detachment following the treatments (Figure 3A; Sheetz, 2001). To avoid human bias, only the last step, i.e., tether breaking force, was selected (Sun et al., 2005; Li et al., 2020). Both MβCD and 3oc caused a significant reduction in tether forces (Supplementary Figure 3), which can be translated into membrane tension per a published formula. In this study, both treatments led to a similar reduction in membrane tension (Figure 3B). While membrane tension can be a property of a pure bilayer, the extensive anchoring mechanisms between the inner leaflet and the cortical cytoskeleton contribute significantly to this physical property as well (Kapus and Janmey, 2013). To rule out this potential caveat, we treated cells with 10 μM 3oc and 10 mM MβCD and chased the cortical skeletal change with Phalloidin. Overall, the impact on the F-actin intensity by both treatments was not profound, the intensity of staining waned slightly at 120 and 60 min for MβCD and 3oc, respectively (Supplementary Figure 4), and this was after the effect on the membrane tension started to appear (∼15 min). In addition, if the force curves produced within the first 30 min after the treatment were compared with those produced in the next 30 min, the overall reduction in tension by both treatments were similar (Supplementary Figure 5), suggesting that the tension change might not rely on the slow-onset cytoskeletal alteration. While we cannot rule out the indirect effect *via* cytoskeletal change induced by MβCD, our results seem to indicate that in the short-term treatments, both MβCD and 3oc can reduce membrane tension.

### Lipid Raft Disruption Affects Cell Signal Transduction and Receptor Endocytosis

**FIGURE 4:**
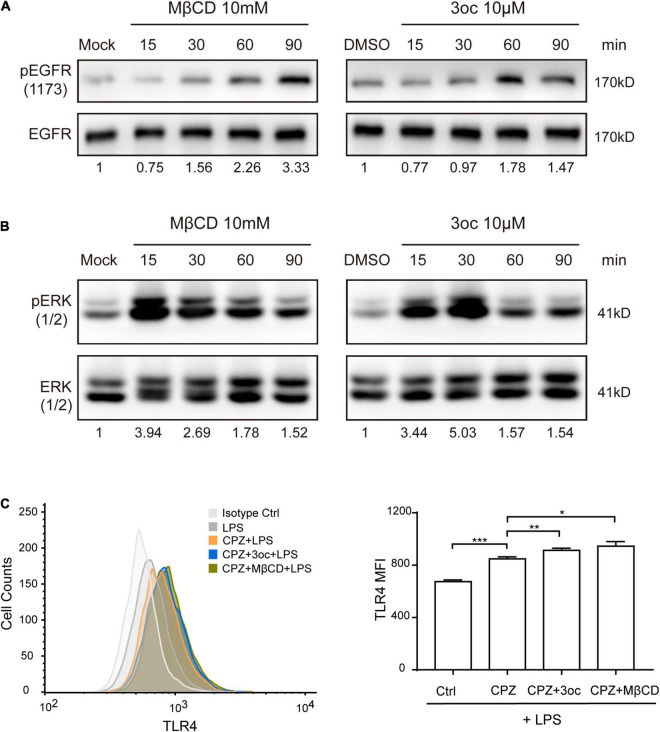
The effects of lipid-ordered domain disruption on cell signal transduction. **(A)** MβCD and 3oc treatments induce spontaneous phosphorylation of epidermal growth factor receptor (EGFR) at tyrosine 1,173 at the indicated time. **(B)** Lipid-ordered domain disruption leads to a similar extent of extracellular signal-regulated kinase (ERK) phosphorylation. **(C)** The disruption of the lipid-ordered domain leads to the obstruction of lipid raft-dependent endocytosis of TLR4, the left panel is the representative histogram of the flow cytometry expression of TLR4 on THP-1 cells treated with LPS (10 μg/ml), LPS + CPZ, LPS + CPZ + 3oc, or LPS + CPZ + MβCD, and the right panel is the quantitative data shown as mean fluorescence intensity ± SEM. The values reported under each blot are densitometry analysis relative to controls taken as 1 after normalization by internal reference. All experiments were repeated three times.

As MβCD and 3oc both disrupted lipid-ordered domains and reduced membrane tension to a similar extent, we wondered if they have similar effects in biological assays with regard to lipid raft disruption. One of the common effects of MβCD cholesterol removal is the spontaneous activation of EGFR; we, therefore, analyzed EGFR phosphorylation at tyrosine 1,173 in the absence of its ligand (Chen and Resh, 2002). Clearly, both treatments led to a substantial increase of pEGFR, suggesting in this assay the two reagents are functionally equivalent (Figure 4A). However, the peak phosphorylation of 3oc treatment took place 30 min earlier than MβCD treatment. Similarly, lipid domain disruption causes spontaneous ERK activation (Kabouridis et al., 2000). Figure 4B shows that both 3oc and MβCD led to a similar extent of ERK phosphorylation. Caveolae are caveolin-dependent, specialized lipid rafts characterized by an inward indented membrane “cave” and an abundance of PIP2 in the corresponding inner leaflets (Fujita et al., 2009). They are a significant source of membrane endocytosis. We first treated THP-1 cells with CPZ, which blocks clathrin-dependent internalization, and then with LPS to trigger caveolae-dependent TLR4 receptor endocytosis (Varkevisser et al., 2013). Flow cytometry results showed that the average fluorescence intensity of TLR4 on the cell membrane increased after treatment, while CPZ treatment inhibited the internalization from 674.3 ± 7.9 to 848 ± 9.6, both MβCD and 3oc treatments further increased the membrane retention of TLR4 from 848 ± 9.6 to 945.7 ± 20 and 913.3 ± 9.3, respectively, suggesting the effective blockage of caveolae function by both treatments (Figure 4C).

### A Low Concentration of 3-Oxododecanoyl Is a Generalizable Raft Modifier Without Altering the Cell Survival

**FIGURE 5:**
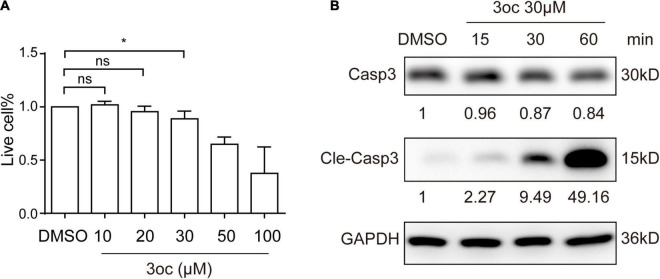
Cell viability of THP-1 cells treated with different concentrations of 3oc. **(A)** Cell apoptosis was detected by the Annexin V/PI apoptosis detection kit and then measured by flow cytometry, statistical analysis results of three experiments. Live cell% = (Annexin V negative cells in MβCD or 3oc treatment/total cells)/(Annexin V negative cells in DMSO treatment/total cells) × 100%. **(B)** The Western blot results show that when the 3oc concentration reaches 30 μM, and it can induce obvious activation of Caspase 3. The values reported under each blot are densitometry analysis relative to controls taken as 1 after normalization by internal reference. All experiments were repeated three times, and the bars represent the mean ± SD (*n* = 3); *P < 0.046 as measured by an unpaired *t*-test.

Unlike non-myeloid cells, immune cells are sensitive to 3oc-induced apoptosis (Song et al., 2019), we wondered if 3oc treatment on THP-1 cells can be precisely controlled to permit lipid domain analysis without triggering significant cell death. As the 3oc concentration used in this report was maintained at 10 μM, we performed a dose-response analysis. In this study, 10 and 20 μM of 3oc did not significantly alter the cell survival, while higher concentrations led to measurable apoptosis at 100 μM (Figure 5A, and the results of the flow cytometry analysis were shown in Supplementary Figure 6). At the higher concentrations, THP-1 cell death was mediated by the canonical Caspase 3 pathway, leading to its cleavage after about 30 min treatment with 3oc at 30 μM (Figure 5B).

## Discussion

Compared with MβCD with its working mechanism proposed decades ago, we currently do not understand how 3oc works. In our previous study (Song et al., 2018), we showed that to disrupt lipid rafts, the acyl chains of homoserine lactones must be no shorter than 10 carbons and require a double-bonded oxygen following the homoserine. The acyl chain length was as expected to correlate with their ability of membrane insertion. The oxo group, in contrast, may be the key to cause the breaking of the semicrystalline structure formed between gangliosides and cholesterol (Simons and Toomre, 2000).

**FIGURE 6:**
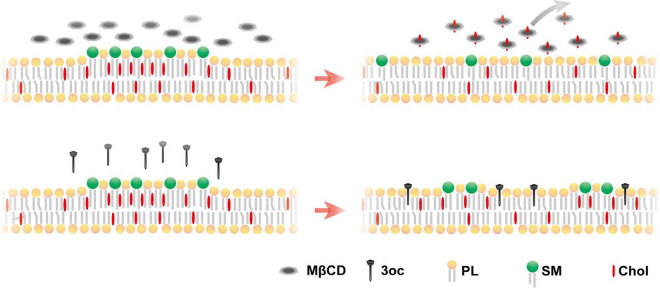
Proposed mechanism for MβCD and 3oc disruption of the lipid-ordered domain. Upper panel: MβCD treatment produces a more homogeneous plasma membrane due to the extraction of lipid components. Lower panel: 3oc produces a more limited disintegration with small islands remaining, without affecting the plasma membrane components [(phospholipids (PLs)].

Our results suggest that 3oc is a viable replacement for MβCD for the purpose of lipid-ordered domain disruption. The 3oc is to some extent more efficient and requires lower effective concentrations, and in some systems, the effect is more rapid. Perhaps the most advantageous feature is the retention of all classes of membrane lipids after the treatment, while MβCD leads to a dramatic reduction in all (Figure 6). The non-specific depletion by MβCD was reported previously, and it was considered as a consequence of high MβCD (Sanchez et al., 2011). Our results suggest that this off-target effect is measurable in common concentrations. 3oc is therefore expected to impact cell biology to a lesser extent. As the studies of lipid-ordered domain almost inevitably use MβCD as the *de novo* treatment, it is possible that some of the effects, particularly those related to receptor signaling on internalization, such as CD95 and insulin receptor (Di Guglielmo et al., 1998; Lee et al., 2006), may not be entirely attributable to lipid domain disruption. Whether 3oc offers a distinct advantage with reduced membrane replenishment therein remains to be fully investigated.

A note of caution is that in some cell types, particularly immune cells, 3oc treatment may induce TNFR1-dependent cell death at high concentrations, which may require an evaluation of the dosage used. In our laboratory, the blockage of Caspase 8 or deficiency of TNFR1 inhibited cell death, which may serve as a reference if 3oc is used on immune cells.

## Data Availability Statement

The original contributions presented in the study are included in the article/Supplementary Material, further inquiries can be directed to the corresponding author/s.

## Author Contributions

HR, CZ, and YX performed all the experiments. XF, TX, and YS conceptualized and designed the experiments. YS wrote the manuscript. All authors contributed to the article and approved the submitted version.

## Conflict of Interest

The authors declare that the research was conducted in the absence of any commercial or financial relationships that could be construed as a potential conflict of interest.

## Publisher’s Note

All claims expressed in this article are solely those of the authors and do not necessarily represent those of their affiliated organizations, or those of the publisher, the editors and the reviewers. Any product that may be evaluated in this article, or claim that may be made by its manufacturer, is not guaranteed or endorsed by the publisher.
