# Supplementary material for: N-(3-Oxododecanoyl) Homoserine Lactone Is a Generalizable Plasma Membrane Lipid-Ordered Domain Modifier
Source: Front Physiol. 2022 Feb 22;12:758458. doi: 10.3389/fphys.2021.758458 (PMC8920551; doi:10.3389/fphys.2021.758458)
Supplement: Supplementary file 1 [file Data_Sheet_1.docx]

Supplementary Material

# Supplementary Figures


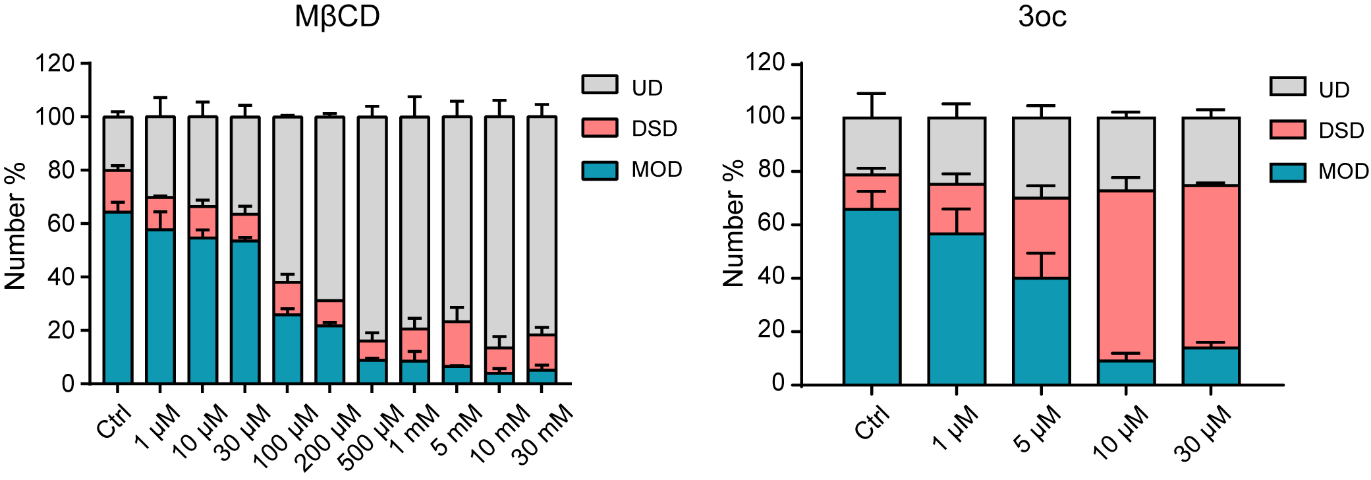


**Supplementary Figure 1.** Comparing the concentration at which MβCD and 3oc can induce lipid domain changes on GPMVs. Concentrations were used as indicated, confocal imaging of all different concentrations was performed after 30 min of MβCD or 3oc treatment.


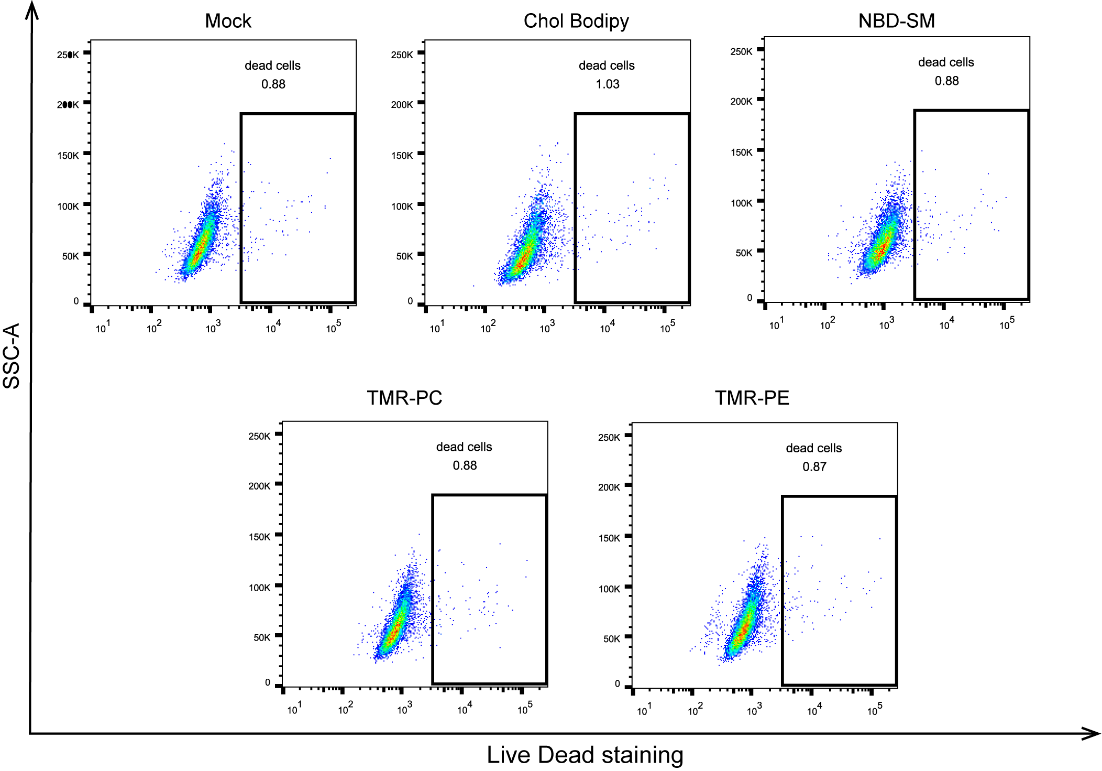


**Supplementary Figure 2.** The addition of fluorescently labeled lipids on the cell membrane did not affect cell viability. 10μg/ml fluorescent labeled lipids Bodipy-Chol, NBD-SM, TMR-PC and TMR-PE were used to incubate the cell for 30 min. The images presenting viability of cells, cells are stained with the live/dead fixable violet stain with 405 nm excitation and then measured by flow cytometry.


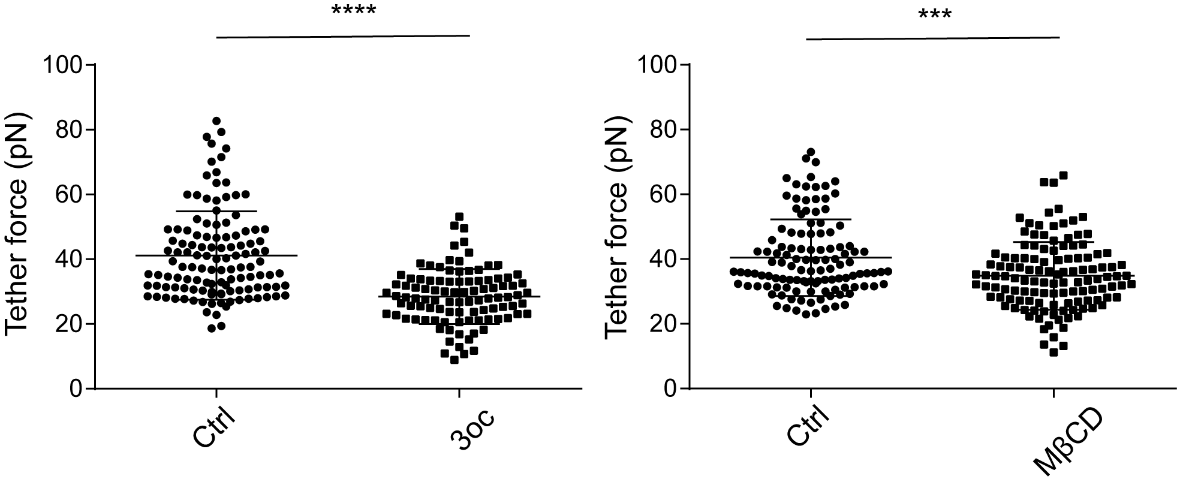


**Supplementary Figure 3.** Tether force of Hela cells in resting state and after the treatment of 3oc and MβCD respectively. Each data point was from one force measurement, and for each cell 3 measurements were performed and about 50 cells were probed for each experimental condition. ****P < 0.0001, ***P < 0.0003 as measured by an unpaired t test.


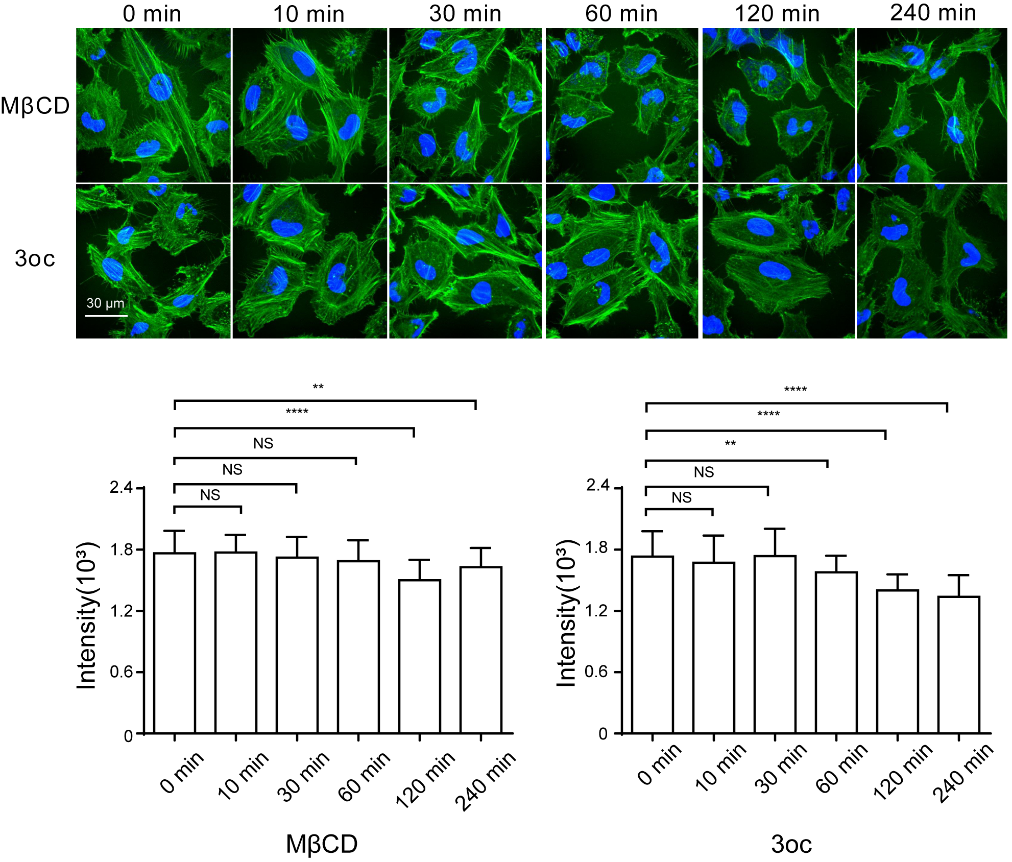


**Supplementary Figure 4.** The effects of 10mM MβCD and 10μM 3oc treatment on cytoskeleton F-actin in Hela cells. The upper panel are confocal images of the cytoskeleton treated with MβCD and 3oc, respectively. The bottom panel are the quantitative data shown as mean fluorescence intensity ± SEM.


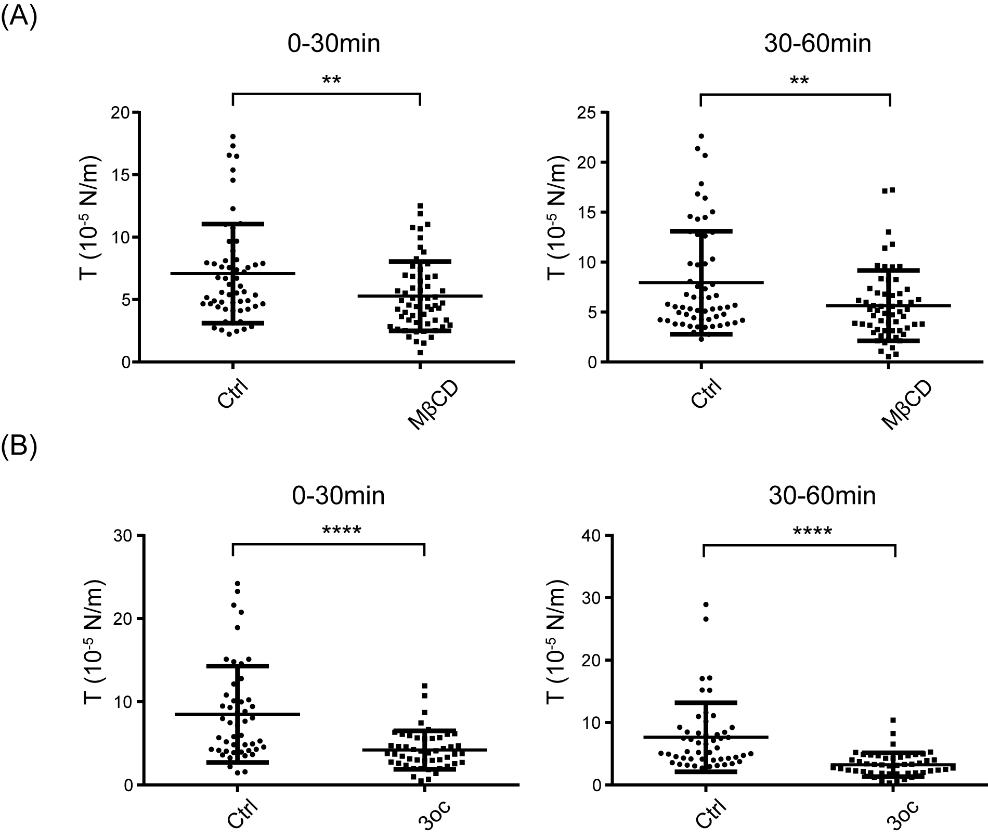


**Supplementary Figure 5.** Membrane tension probed by AFM-based tether force measurements. (A) Membrane tension of Hela cells in resting state and after the treatment of MβCD, the left panel is the membrane tension produced within the first 30 min after the treatment, the right panel is the membrane tension of 30-60 min after the treatment. (B) Membrane tension of Hela cells in resting state and after the treatment of 3oc, the left panel is the membrane tension produced within the first 30 min after the treatment, the right panel is the membrane tension of 30-60 min after the treatment.


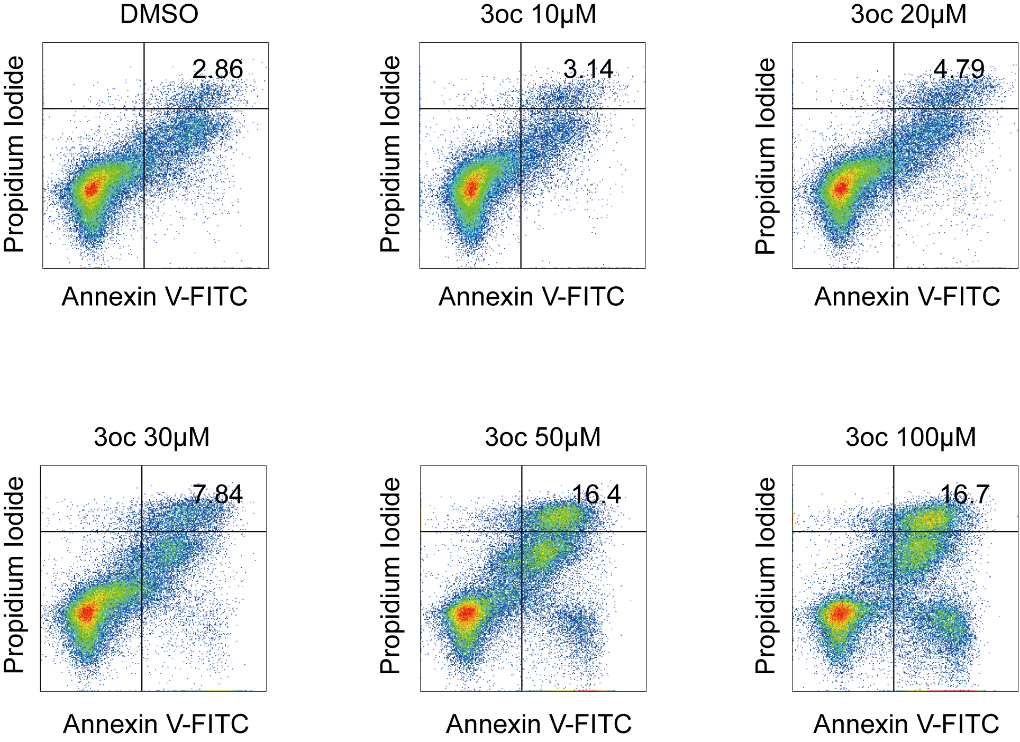


**Supplementary Figure 6.** Cell apoptosis was detected by the annexin V/PI apoptosis detection kit and then measured by flow cytometry.

# Uncropped Gels


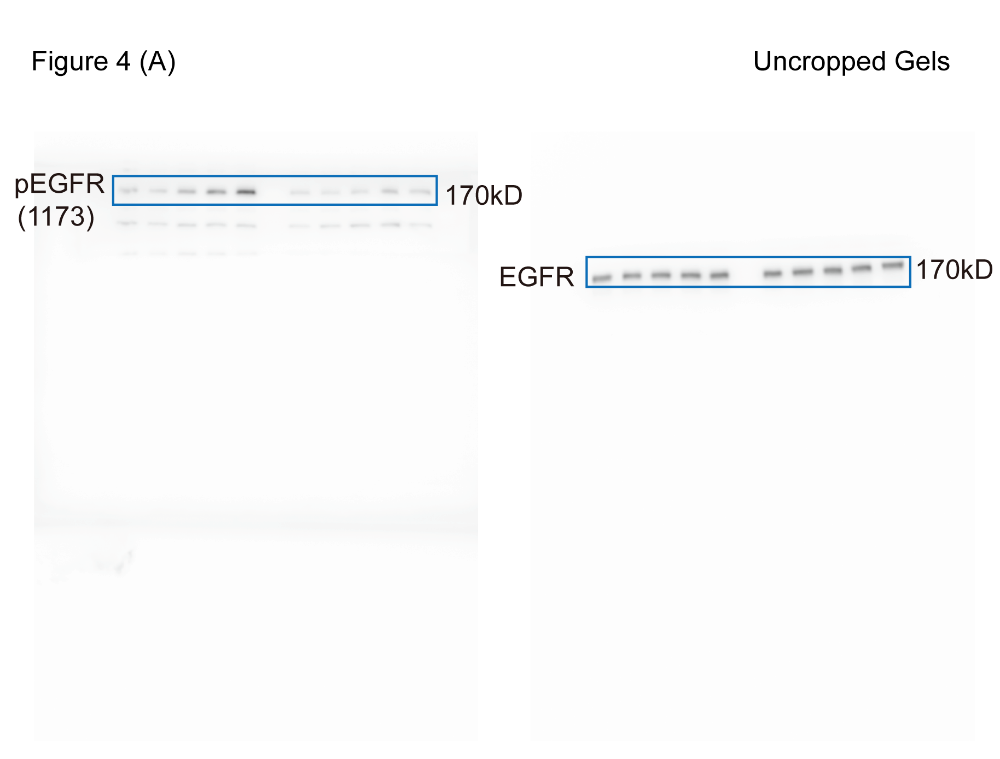


**Figure 4A.** Uncropped gels of EGFR phosphorylation in **Figure 4**.


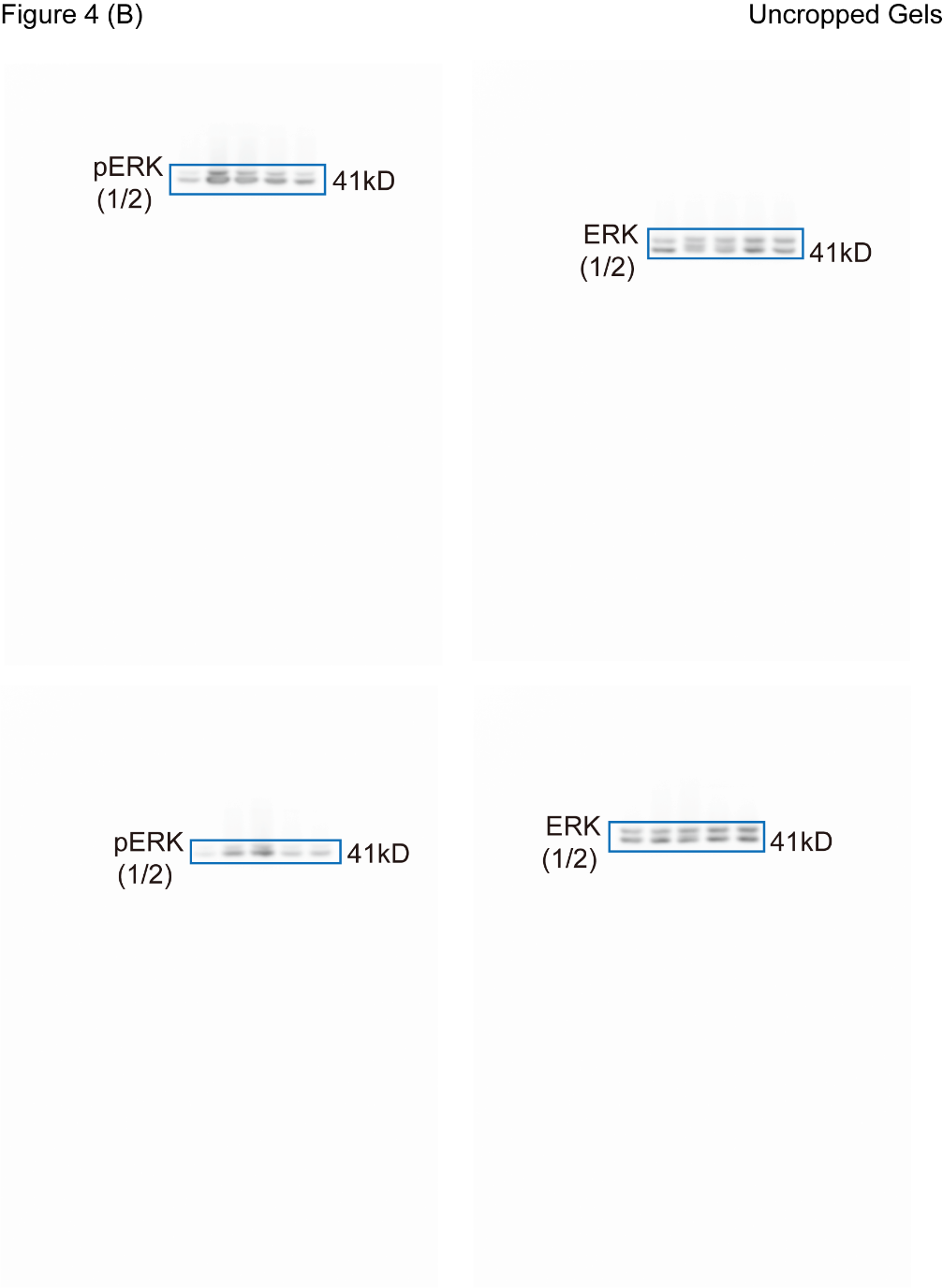


**Figure 4B.** Uncropped gels of ERK phosphorylation in **Figure 4**.


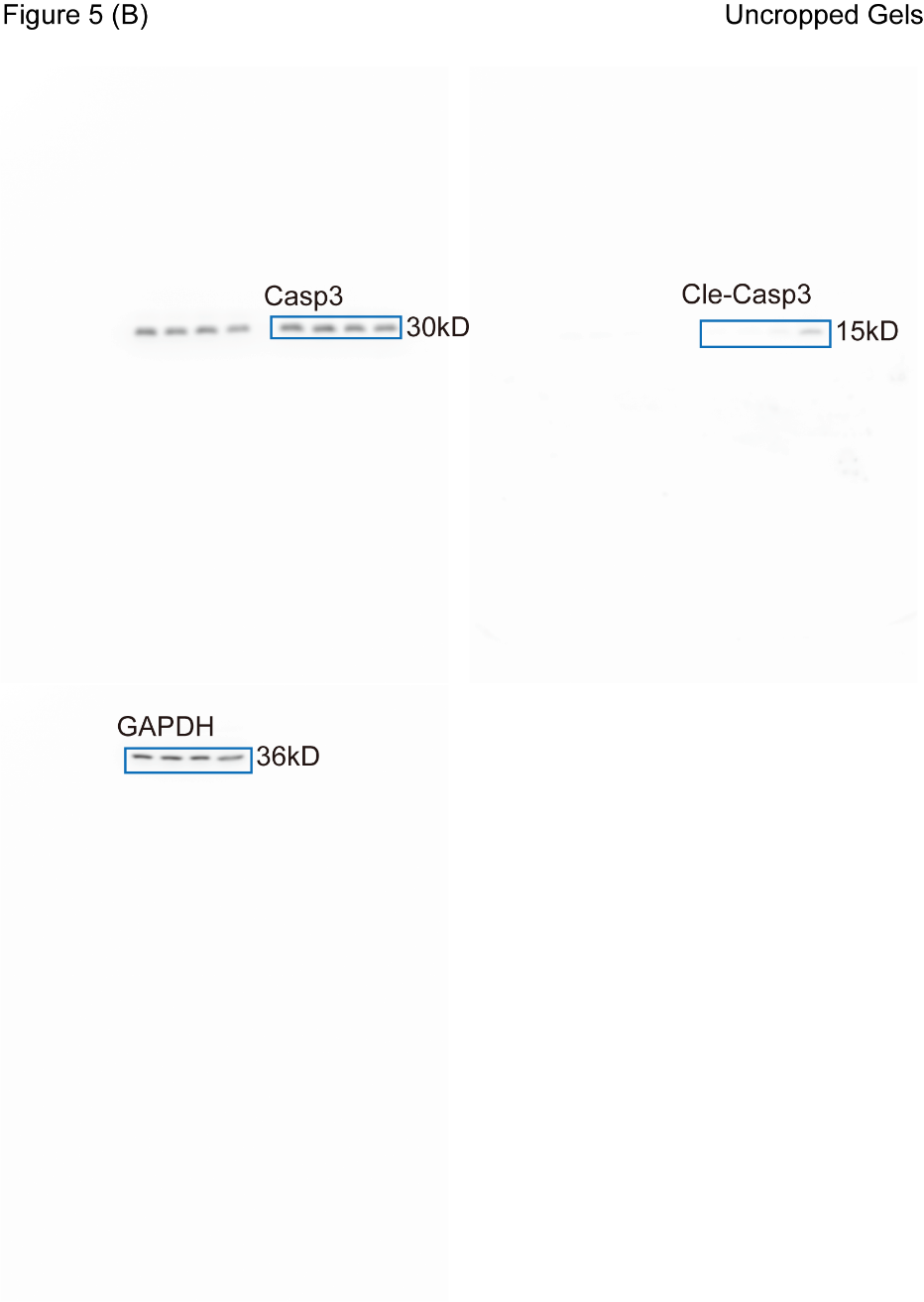


**Figure 5B.** Uncropped gels of ERK phosphorylation in **Figure 5**.
